# Allergic diseases of the skin and drug allergies – 2021. Efficacy of montelukast as add-on therapy in patients with chronic idiopathic urticaria

**DOI:** 10.1186/1939-4551-6-S1-P108

**Published:** 2013-04-23

**Authors:** Sujoy Khan, Nuala Lynch, Sujoy Khan

**Affiliations:** 1 Department of Immunology, Frimley Park Hospital NHS Foundation Trust, Frimley, UK; 2 Hook Surgery, Hook, UK

## Background

Treatment of chronic idiopathic urticaria (CIU) can be difficult with antihistamines only and therefore we assessed (1) efficacy ofmontelukast as add-on therapy, and (2) if any clinical features or laboratory markers were associated with a response to montelukast.

## Methods

Patients who received montelukast for CIU between 2008-2011 (4-year period) were retrospectively identified from clinic letters. Clinical features of duration of urticaria, medication use, autoimmunity and laboratory investigations that included basopenia and mean platelet volume on full blood count, complement levels, specific IgE, autoimmune serology [antinuclear antibody (ANA), thyroid peroxidase antibodies, serum histamine releasing antibody (using donor basophils, positive >18.5%)] were collected and analysed. The primary end point was adequate control of urticaria without additional therapy. Patients with features of urticarial vasculitis and those who required corticosteroids or immunosuppressants were excluded. Nonparametric statistical data were calculated using GraphPad Prism software.

## Results

28 patients (11 males, 17 females; age mean±SD 36.5±15.4 years) received montelukast and the average duration of urticaria was 3.5 years. Six patients had autoimmunity (5 diabetes, 1 hypothyroidism). 24 patients (86%) were on anti-H1 (cetirizine 10mg/fexofenadine 180mg) and anti-H2 blockers (ranitidine 150mg) when montelukast was started. 13 patients (46.4%) responded to montelukast; the mean±SD age 31±13.98 years of 9 males and 4 females (age mean±SD 35±14.99 years) who responded was non-significant (2-sided unpaired *t* test p value 0.6498). Duration of anti-H1 use (<1 year vs >1 year) between males/females and response to montelukast was also non-significant (p value 0.4887). One patient responded to montelukast and fexofenadine only. Two patients required short course of steroids for urticarial flare while on montelukast. 9 patients continue to remain on montelukast. 17 patients had basopenia, 9 high mean platelet volumes, 2 had positive ANA, 4 positive specific IgE, C3/C4 levels tested in all 23 patients were normal, 4 had positive histamine release antibody test and 3 with thyroid peroxidase antibodies. 6 patients had montelukast related side effects: nausea (1), dyspepsia (1), depression (1) and worsening urticaria (3).

## Conclusions

Montelukast as add-on therapy with anti-H1/anti-H2 blockers was effective in almost half of patients with CIU. No clinical features or laboratory markers were associated with response to montelukast.

